# Multi-modal features-based human-herpesvirus protein–protein interaction prediction by using LightGBM

**DOI:** 10.1093/bib/bbae005

**Published:** 2024-01-26

**Authors:** Xiaodi Yang, Stefan Wuchty, Zeyin Liang, Li Ji, Bingjie Wang, Jialin Zhu, Ziding Zhang, Yujun Dong

**Affiliations:** Department of Hematology, Peking University First Hospital, Beijing, China; Department of Computer Science, University of Miami, Miami FL, 33146, USA; Department of Biology, University of Miami, Miami FL, 33146, USA; Institute of Data Science and Computation, University of Miami, Miami, FL 33146, USA; Sylvester Comprehensive Cancer Center, University of Miami, Miami, FL 33136, USA; Department of Hematology, Peking University First Hospital, Beijing, China; Department of Hematology, Peking University First Hospital, Beijing, China; Department of Hematology, Peking University First Hospital, Beijing, China; Department of Hematology, Peking University First Hospital, Beijing, China; State Key Laboratory of Animal Biotech Breeding, College of Biological Sciences, China Agricultural University, Beijing 100193, China; Department of Hematology, Peking University First Hospital, Beijing, China

**Keywords:** human-herpesvirus interaction, protein–protein interaction, multi-modal, embedding, LightGBM, prediction

## Abstract

The identification of human-herpesvirus protein–protein interactions (PPIs) is an essential and important entry point to understand the mechanisms of viral infection, especially in malignant tumor patients with common herpesvirus infection. While natural language processing (NLP)-based embedding techniques have emerged as powerful approaches, the application of multi-modal embedding feature fusion to predict human-herpesvirus PPIs is still limited. Here, we established a multi-modal embedding feature fusion-based LightGBM method to predict human-herpesvirus PPIs. In particular, we applied document and graph embedding approaches to represent sequence, network and function modal features of human and herpesviral proteins. Training our LightGBM models through our compiled non-rigorous and rigorous benchmarking datasets, we obtained significantly better performance compared to individual-modal features. Furthermore, our model outperformed traditional feature encodings-based machine learning methods and state-of-the-art deep learning-based methods using various benchmarking datasets. In a transfer learning step, we show that our model that was trained on human-herpesvirus PPI dataset without cytomegalovirus data can reliably predict human-cytomegalovirus PPIs, indicating that our method can comprehensively capture multi-modal fusion features of protein interactions across various herpesvirus subtypes. The implementation of our method is available at https://github.com/XiaodiYangpku/MultimodalPPI/.

## INTRODUCTION

Herpesviruses are ubiquitous and latently transmitted in eukaryotes. Herpesvirus infections are usually mild, but can lead to severe diseases such as encephalitis, birth defects of sensory nerves and tumors in patients with weak immune responses [1]. In particular, herpesvirus infection and reactivation commonly occur during the development of multiple malignancies such as hematological malignancies [2–4]. As a consequence of weak immune responses in populations of e.g. hematopoietic stem cell transplantation patients, reactivation of herpesviruses may cause a variety of organ dysfunction (e.g. respiratory failure caused by cytomegalovirus pneumonia) [5], which may impair the final curative effect of tumor treatments. Human herpesviruses (HHV) can be divided into three categories, such as alpha [HHV-1, HHV-2, varicella-zoster virus (VZV)/HHV-3], beta [cytomegalovirus (CMV/HHV-5), HHV-6A, HHV-6B and HHV-7], gamma [Epstein–Barr virus (EBV/HHV-4) and Kaposi’s sarcoma-associated herpesvirus (KSHV/HHV-8)] subfamilies. Except for HHV-1, VZV and CMV, there is currently no effective antiviral drug or vaccine for herpesvirus infections [6, 7]. Herpesvirus infection and host immune response are largely determined by human-herpesvirus protein–protein interactions (PPIs) [8, 9]. Therefore, the systematic characterization and analysis of human-herpesvirus PPIs is essential for our in-depth understanding of the pathogenic mechanisms of herpesvirus infection and development of anti-herpesvirus drugs, effectively improving the prognosis of hematological tumors.

High-throughput experimental techniques such as yeast two-hybrid and affinity purification mass spectroscopy have identified a substantial number of human-herpesvirus PPIs [10–12]. However, such interaction data were mainly found in EBV [10, 11], CMV [12], KSHV [13, 14] and HSV-1 [15, 16], while PPIs of other herpesvirus subtypes are under-investigated. As a consequence, it is paramount to identify interactions, that provide the basis to elucidate mechanism differences of interactions between human host and different herpesvirus subtypes. As the large-scale experimental determination of PPIs is usually time-consuming and laborious, efficient computational prediction methods can complement experimental methods to provide testable protein pairs with high confidence.

Numerous computational methods have been previously developed to predict protein interactions including interolog mapping [17], domain–domain/motif interaction-based inference [18] and structure-based prediction methods [19]. Moreover, machine learning (ML) and artificial intelligence (AI) approaches have also been employed to predict PPIs, usually pointing to superior performance compared to more traditional non-ML methods. Although ML methods have been predominantly applied to predict intra-species PPIs, a series of inter-species ML-based human–virus PPI prediction methods have been proposed [20–23]. However, ML-based PPI prediction methods that were specifically designed for the prediction of interactions between proteins of human and herpesviruses are still limited. To our best knowledge, only Lian *et al*. developed an in silico prediction method that incorporated interolog mapping, domain–domain interaction-based inference and ML to predict human-herpesvirus PPIs [24]. Only focusing on human-HSV-1, they trained a random forest (RF) model that integrated traditional CKSAAP sequence features and six network parameters, where the feature encoding schemes and ML algorithm applied were simple and rather insufficient to represent protein features and to yield favorable prediction performance. Moreover, some previous human–virus PPI prediction models trained on all human–virus interactions, which may potentially lack specificity for herpesvirus.

ML-based prediction methods are mainly based on two core steps, capturing feature encoding and model training. Efficient feature encoding methods fully reflect latent features of samples and improve model learning efficiency. Commonly used encoding methods are currently based on sequence information such as di-peptide composition (DPC) [25], conjoint triad (CT) [26] and auto covariance (AC) [27], capturing amino acid composition, chemical properties or residue interaction effects. With the development of AI methods, several natural language processing (NLP)-driven embedding methods have been applied to predict PPIs [28]. Specifically, Word2vec is an NLP-driven word embedding technique, that adopts shallow neural networks to obtain feature vectors of sequence k-mers [29]. For example, Tsukiyama *et al*. employed Word2vec to learn embeddings of amino acid k-mers (i.e. words) in protein sequences (i.e. sentences) to predict human–virus PPIs [22]. As an extension of Word2vec, Doc2vec captures the whole sentence as another method to learn embeddings, considering context information of the words and the whole sentence. In particular, our previous work [20] used Doc2vec to obtain embedding feature vectors of protein sequences (i.e. sentences) to predict human–virus PPIs. Furthermore, the graph embedding technique node2vec has been widely used to represent nodes and edges in biological graphs to classify nodes or predict edges. In particular, Node2vec uses random walks to generate node sequences from the graph, which are further fed into the Word2vec model to find node feature representations. In particular, Liu-Wei *et al*. employed a node2vec variant DL2Vec to embed human and viral proteins through a Gene Ontology (GO) network and disease phenotype annotations to predict human–virus PPIs [23].

As for model training, various ML algorithms such as RF, support vector machine (SVM) and convolutional neural networks (CNN) have been used to predict human–virus PPIs with favorable prediction performance. In recent years, an ensemble ML algorithm light gradient boosting machine (LightGBM) showed impressive performance in predicting secreted effectors, residue binding sites and drug-target interactions [30–32], prompting us to introduce LightGBM to predict human-herpesvirus PPIs. Specifically, we based LightGBM on multi-modal embeddings of sequences, networks and functions to predict human-herpesvirus PPIs, and evaluated our model’s performance through non-rigorous and rigorous partitions of training and test data that we constructed from human-herpesvirus PPI datasets. Our results clearly suggested that our multi-modal features-based integration model shows superior performance by significantly providing better results than single-modal features-based models, various ML models based on different traditional sequence encodings and existing state-of-the-art human-virus PPI prediction methods.

## MATERIALS AND METHODS

**Figure 1:**
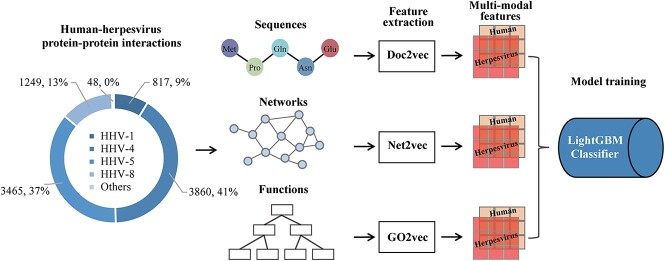
Workflow of human-herpesvirus PPI prediction based on multi-modal features (i.e. sequences, networks and functions). Utilizing interactions between proteins of different herpesvirus subtypes and human, we employed different feature extraction methods, pertaining to protein sequences, interactions between proteins and protein functions. In particular, we extracted feature embeddings of protein sequences through Doc2vec, while we represented interacting proteins through Node2vec and functions through GO2vec to generate multi-modal features of the underlying PPI. Finally, we trained our LightGBM classifier on the merged features to predict the presence/absence of an interaction between a human and a viral protein.

A schematic flow chart of our proposed method is shown in Figure 1. First, we collected protein sequence information, known interaction networks and gene functional annotations from UniProt [33], IntAct [34], BioGRID [35], VirHostNet [36], VirusMentha [37] and GO [38] databases. Subsequently, we employed different feature extraction methods based on protein sequences, networks and functions to generate multi-modal features to train our LightGBM classifier on the merged features.

### Dataset construction

We collected human-herpesvirus PPI data from five public molecular interaction databases, including IntAct [34], BioGRID [35], HPIDB [39], VirHostNet [36] and VirusMentha [37]. In the next filtering step, non-physical interactions, redundant interactions and interactions between proteins with less than 30 amino acids, more than 5000 amino acids or non-standard amino acids were removed, resulting in 9439 positive sample PPIs. Specifically, our positive samples involved seven HHVs except HHV-7 (i.e. HHV-1 ~ HHV-6 and HHV-8) (Figure 1).

As for negative sample selection, we considered viral and human proteins that also appeared in the positive training data sets and human proteome. In particular, we used the ‘Dissimilarity-Based Negative sampling’ [20, 40] method to reduce the introduction of false negative samples. Specifically, we inferred a protein pair B-C as a potential interaction that was not selected as a negative sample if viral proteins A and B were similar (sequence identity >0.3), assuming that human protein C interacted with viral protein A.

Furthermore, previous studies [27, 41, 42] indicated that the predictive performance of pair-input methods may be overestimated as a consequence of shared proteins in training and test sets. In other words, proteins have a higher chance to be classified as interacting as a consequence of their overrepresentation in the training data. To avoid such a bias, we employed a rigorous sampling strategy [27, 41] to find negative training data. Considering 80% of known viral-human PPIs as positive samples while the remainder served as test set, we randomly sampled 10 times as many negative than positive samples to obtain 94,390 negative samples. In particular, we ensured that viral proteins in the test set were sequence-dissimilar (sequence similarity <0.3) compared to the viral proteins in the positive training samples (i.e. rigorous partition). Moreover, we also provided non-rigorous training data, by randomly sampling a training and test set, where viral proteins in the test set were similar compared to the training set. Furthermore, we used three sets of replicates per sampling strategy to assess the model’s performance.

### Feature encodings

#### Doc2vec

We used the document embedding technique Doc2vec to represent the context semantic features of protein sequences that were treated as sentences written in a certain biological language. In particular, protein sequences constitute a ‘document’ (i.e. a corpus) and convey biological functions and meanings that can be semantically interpreted through the Doc2vec model [43]. First, each amino acid sequence (i.e. sentence) was broken into k-mer fragments (i.e. words). Subsequently, k-mers of amino acids and the complete sequence were used to train the Doc2vec model, allowing us to obtain a fixed-dimensional feature vector for each protein sequence. Here, we focused on non-redundant protein sequences from our protein interaction samples and SwissProt database where we used CD-HIT to remove redundancy by considering sequence identity of ≤0.5 [44] as corpus of the Doc2vec model training. In our previous work [20], we applied three methods of k-mer extraction [43, 45]: For instance, the sequence ‘MPQNEY’ was broken into 2-mers such as [‘MP’, ‘PQ’, ‘QN’, ‘NE’, ‘EY’]; [‘MP’, ‘QN’, ‘EY’], [‘PQ’, ‘NE’]; [‘MP’, ‘QN’, ‘EY’, ‘PQ’, ‘NE’] where the latter extraction method performed best in predicting human–virus PPIs. Furthermore, we augmented such k-mers with single amino acid residues (i.e. k = 1). Based on our previous works [20, 46], we set the baseline parameters i.e. ‘extraction_method’ = 3, *k* = 5, ‘vector_size’ = 32, window = 3 and epoch = 70 to optimize them one by one.

We used the python library Gensim [47] to train the Doc2vec model and adopted the distributed-memory (DM) model architecture of Doc2vec [29], allowing us to characterize each amino-acid k-mer through a vector of context-specific k-mers and the complete protein sequence vector. Using stochastic gradient descent and backpropagation to update the weight parameters of the model, we optimized parameters (e.g. k-mers, window size and the dimensionality of output vectors) by 5-fold cross-validation based on the non-rigorous sampling datasets. For each Doc2vec parameter combination, we trained three LightGBM models on the three replicates of non-rigorous sampling datasets by using the extracted feature vectors of Doc2vec. We obtained optimal parameter combinations of Doc2vec models through averaging the performance over all three LightGBM models.

#### Net2vec

Intra-species protein interaction networks were constructed to characterize network properties of proteins. First, we collected human protein interactions and herpesvirus protein interactions from four public protein interaction databases such as IntAct [34], BioGRID [35], VirHostNet [36] and VirusMentha [37]. In total, we obtained 329 611 human PPIs between 26 691 human proteins and 2104 herpesviral PPIs between 706 herpesviral proteins after removing redundant and genetic interactions. In the next step, we trained the Node2vec model on the human PPI network and herpesvirus PPI network, respectively. Multiple node sequences were generated through the random walk process, which were further fed to the Word2vec model to obtain protein node feature vectors. We calculated the average feature vectors of all proteins in the human PPI network and the herpesvirus PPI network, respectively. The average feature vector of human/herpesvirus proteins was used to characterize the human/herpesvirus proteins that were not present in human/herpesvirus intra-species PPI networks. Here, we set the parameters ‘walk length’, ‘numbers of walks’ and the size of output feature vector of Net2vec model training to 30, 200 and 32, respectively.

#### GO2vec

We utilized the GO hierarchical network and GO annotation information to represent the functional properties of human and herpesvirus proteins. GO hierarchical relationship and GO annotation data of human and herpesvirus proteins were downloaded from the GO database (http://geneontology.org/). Subsequently, we constructed two comprehensive networks containing multiple nodes (i.e. GO terms and proteins) and edges (i.e. GO term-GO term and protein-GO term) for human and herpesvirus, respectively. Similar to the Net2vec encoding scheme, Node2vec was employed to obtain node embedding features of GO terms and proteins in the network. When an encoded protein was not in the GO term-GO term/protein network, we assigned the average vector of the network protein nodes. Here, we set the parameters ‘walk length’, ‘numbers of walks’ and the size of output feature vector of GO2vec model training to 30, 200 and 64, respectively.

### LightGBM classifier

LightGBM is an ensemble model based on decision trees for solving various classification and regression problems. Specifically, weak classifiers (decision trees) are iteratively trained to get the optimal model, that lead to satisfactory training effects and avoid overfitting. LightGBM is an improved extension of the gradient boosting decision tree [48], which employs gradient-based one-side sampling (GOSS) and exclusive feature bundling (EFB) [49]. GOSS reduces the sample dimension by sampling with small gradients, while EFB bundles mutually exclusive features into one novel feature thereby reducing feature dimensions. As a consequence, LightGBM provides fast model training, satisfactory high accuracy and classification/regression generalizability. LightGBM was implemented through the Python-based ML library scikit-learn. Here, we chose ‘binary’ as the learning objective and employed the GridSearchCV function to optimize ‘learning rate’ and ‘max_depth’, capturing optimization ranges of [0.001,0.01,0.05,0.1,0.15,0.2,0.25,0.3] and [10,50,100,200].

### Baseline methods

#### Baseline encoding approaches

As baselines, we encoded sequences through three typical sequence-based encoding methods [25–27]: (i) DPC reflected the ratio of two subsequent amino acid residues in the sequence through ${F}_{DPC}\left({\mathrm{a}}_i,{\mathrm{a}}_j\right)=\frac{N_{a_i{a}_j}}{\mathrm{L}-1}$. ${\mathrm{a}}_i,{\mathrm{a}}_j$ represented 2 of the 20 standard amino acids, while ${N}_{a_i{a}_j}$ and *L* were the number of certain di-peptide in the sequence and the sequence length, respectively. As a result, each protein pair was encoded by an 800 (20 × 20 × 2)-dimensional feature vector. (ii) CT characterized the physicochemical features of amino acids in the sequence through the ratio of a triplet of continuous amino acid classes in the sequence. Specifically, 20 standard amino acids were classified into seven groups (AGV, C, DE, EILP, HNQW, KMSTY and KR) according to their physicochemical properties, providing a 686 (7 × 7 × 7 × 2)-dimensional feature vector for each protein pair. (iii) AC considers the interaction effect between amino acid variables at different positions. Here, seven physicochemical properties, i.e. hydrophobicity (H1), hydrophilicity (H2), polarity (P1), polarizability (P2), solvent accessible surface area, net charge index of side chains and volume of side chains (V) were employed to represent protein features. In particular, the AC score *S_AC_* was defined as ${S}_{AC}\left( lag,j\right)=\frac{1}{L- lag}{\sum}_{i=1}^{L- lag}\left({\mathrm{R}}_{i,j}-\frac{1}{L}{\sum}_{k=1}^L{\mathrm{R}}_{k,j}\right)\times \left({\mathrm{R}}_{\left(i+ lag\right),j}-\frac{1}{L}{\sum}_{k=1}^L{\mathrm{R}}_{k,j}\right),\ j\in \left(1,2,\dots, 7\right)$, where *i* and *k* represented the *i*^th^ and *k*^th^ amino acid residue in the protein sequence while *j* was one of the seven physicochemical features. R*_i,j_* and R*_k,j_* represented the *j*th physicochemical feature of the *i*^th^ and *k*^th^ amino acid residue. *lag* was the distance between the *i*th residue and its adjacent residue, in which *lag* was set to 30. Finally, a 420 (30 × 7 × 2)-dimensional feature vector was obtained for each protein pair.

#### Baseline ML algorithms

RF and SVM are two classical ML algorithms that have been widely used in various binary classification tasks. Furthermore, we also employed a deep learning architecture i.e. multiple layer perceptron (MLP) as another baseline algorithm to compare. These ML algorithms were implemented through the Python-based ML library scikit-learn and deep learning library keras. For all ML algorithms, we optimized parameters through cross-validation sets by utilizing the GridSearchCV function.

### Performance assessment

Two benchmarking datasets (i.e. rigorous and non-rigorous partitions) were used to evaluate the performance of all models. In particular, we trained and tested three models based on three training/test set partitions for each benchmarking dataset and calculated the average performance of three models as final performance check. In particular, we employed 5-fold cross-validation by using 80% as training data to optimize the parameters of the models while the remaining 20% were test data to evaluate the performance of different models. Two commonly used curves were plotted to intuitively show the prediction performance of models such as the receiver operating characteristic (ROC) curve and precision-recall (PR) curve through the areas under the ROC (AUROC) and PR curve (AUPRC) metrics. All ROC and PR curves and metrics were determined through the R package ROCR. In addition, we also introduced four common performance metrics such as:


$$ precision=\frac{TP}{\mathrm{TP}+\mathrm{FP}} $$

$$ recall=\frac{TP}{\mathrm{TP}+\mathrm{FN}} $$

$$ accuracy=\frac{TP+ TN}{\mathrm{TP}+\mathrm{TN}+\mathrm{FP}+\mathrm{FN}} $$

$$ F1=\frac{2\times precision\times recall}{\mathrm{precision}+\mathrm{recall}} $$

where TP, FP, TN and FN represent the number of true positives, false positives, true negatives and false negatives, respectively.

### Enrichment analysis

To find functional and pathway enrichments of the identified herpesviral targets, we downloaded GO annotation data of human proteins from http://current.geneontology.org/ [38]. Moreover, the KEGG pathway data were downloaded from https://www.genome.jp/kegg/ [50]. Using all human proteins mapped to Cellular Component, Biological Process and Molecular Function ontologies as well as all human proteins in all KEGG pathways as reference sets, GO terms and KEGG pathways were deemed significantly enriched with human targets of each herpesvirus subtype through hypergeometric tests, if the corresponding Benjamin-Hochberg corrected *P-*values was ≤0.05.

## RESULTS

### Performance of Doc2vec encoding-based LightGBM classifier

**Figure 2:**
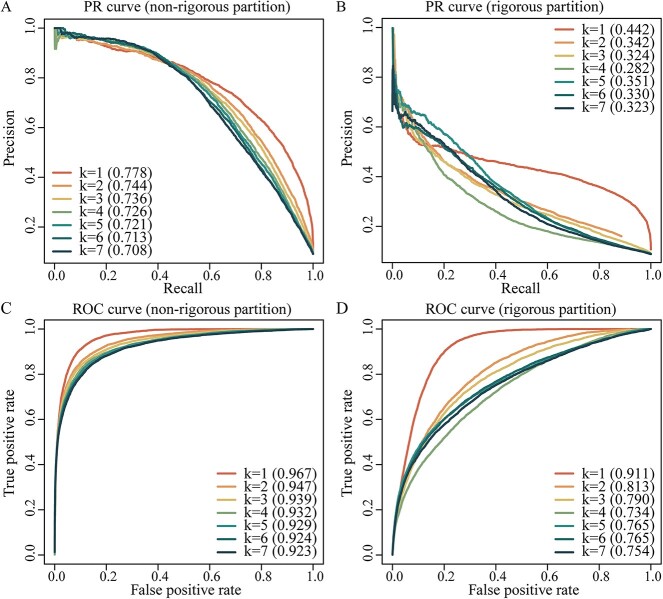
Performance of Doc2vec encoding-based LightGBM models in predicting human-herpesvirus PPIs based on different k-mers of amino acids. Areas under the precision-recall curves (AUPRC) and the areas under the receiver operating characteristic curves (AUROC) indicated that 1-mers effectively improved the performance of the LightGBM. (**A**) PR curves obtained with the non-rigorous partition benchmarking dataset and (**B**) the rigorous partition benchmarking dataset. (**C**) ROC curves obtained with the non-rigorous partition benchmarking dataset and (**D**) the rigorous partition benchmarking dataset.

As the sequence-based Doc2vec embedding encoding technique has a robust performance in PPI prediction, we used Doc2vec to encode protein sequences and obtained corresponding sequence feature as one of our multi-modal features. Furthermore, we used such single-modal features to train the LightGBM classifier with two benchmarking datasets (i.e. non-rigorous and rigorous partitions) of human-herpesvirus PPIs. We utilized 5-fold cross-validation to optimize parameters (i.e. extracted method, k-mers, window size, epoch, and vector size) of the Doc2vec model by comparing corresponding AUPRCs of the LightGBM models. As the optimization baseline for extraction method, *k*, window size, vector size, and epoch we chose 3, 5, 3, 32, and 70, respectively, showing relatively superior performance in our previous work. To optimize such parameters separately, we kept baseline values of the remaining parameters constant. For instance, we tested k-mer sizes 1 to 7 while keeping the unchanged baseline values of other parameters. While ignored in previous studies, we found that 1-mers (i.e. k-mer list: [‘M’, ‘P’, ‘Q’, ‘N’, ‘E’,’ Y’], sequence: ‘MPQNEY’) significantly improved the prediction performance of our model (Table 1 and Figure 2), where AUROC/AUPRC values were 2/3.4 and 9.8/9.1 percentage points higher compared to the best performing k > 1-mers extraction methods. As a result, the combination of Doc2vec with 1-mers, window size 3, vector size 256 and 70 epochs, and LightGBM (Doc2vec + LightGBM) provided the best performance where the corresponding AUROC/AUPRC values were 0.968/0.774 in the non-rigorous and 0.975/0.810 in the rigorous data set using 5-fold cross-validation. In comparison, Doc2vec + RF only achieved AUROC/AUPRC values of 0.973/0.796 (non-rigorous) and 0.924/0.497 (rigorous), respectively.

**Table 1:** 5-fold cross-validation of LightGBM models using different k-mers of amino acids in the Doc2vec encoding scheme

Partition	Metric	k-mers						
		*k* = 1	*k* = 2	*k* = 3	*k* = 4	*k* = 5	*k* = 6	*k* = 7
Non-rigorous	AUROC	0.962	0.939	0.928	0.921	0.916	0.910	0.907
	AUPRC	0.753	0.720	0.707	0.699	0.690	0.683	0.675
Rigorous	AUROC	0.970	0.954	0.945	0.939	0.937	0.932	0.930
	AUPRC	0.792	0.765	0.747	0.738	0.735	0.719	0.716

### Performance of single-modal and multi-modal based LightGBM classifiers

**Figure 3:**
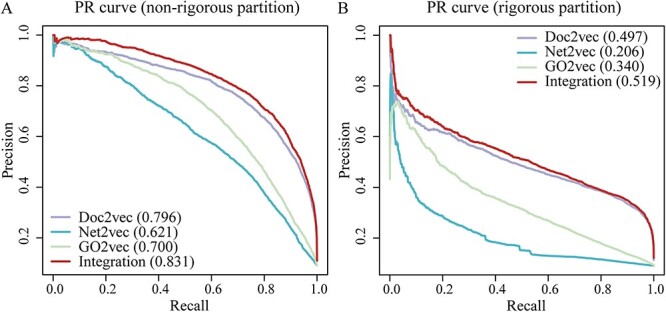
Performance of LightGBM models in predicting human-herpesvirus PPIs based on single-modal (i.e. sequence, network and function) and multi-modal integration features. AUPRC indicated that the multi-modal integration-based LightGBM outperformed different single-modal based LightGBM models. In (**A**), we used the non-rigorous partition benchmarking dataset, while in (**B**) we utilized the rigorous partition benchmarking dataset to evaluate performance.

In addition to sequence-based single-modal features, we also employed two other modals such as network and function characterizations to train separate single-modal feature-based LightGBM models and subsequently concatenated the feature vectors of the three single-modals. To benchmark the performance of three single-modal (i.e. sequence. network and function) and multi-modal integration methods, we performed 5-fold cross-validation with the non-rigorous and rigorous partition benchmarking datasets. As the ratio of positive to negative training sets is highly unbalanced (1:10), we mainly assessed the corresponding performance of our models through analyses of the AUPRC. Generally, we observed that the sequence-based single-modal method outperformed network-based and function-based single-modal models, while the multi-modal integration LightGBM classifier generally outperformed single-modal based models (Figure 3). In particular, we observed that the AUPRC of multi-modal based LightGBM (Integration) was 3.5 and 2.2 percentage points higher compared to the second best performing single-modal method (Doc2vec) (Figure 3), when we considered the non-rigorous and rigorous datasets, respectively. Notably, the sequence-based Doc2vec single-modal encoding scheme showed a relatively modest decline of performance from non-rigorous partition benchmarking to rigorous partition benchmarking compared to the other two single modals capturing network and function, implying that sequence information was still the most informative feature.

### Performance comparison with the baseline methods

**Figure 4:**
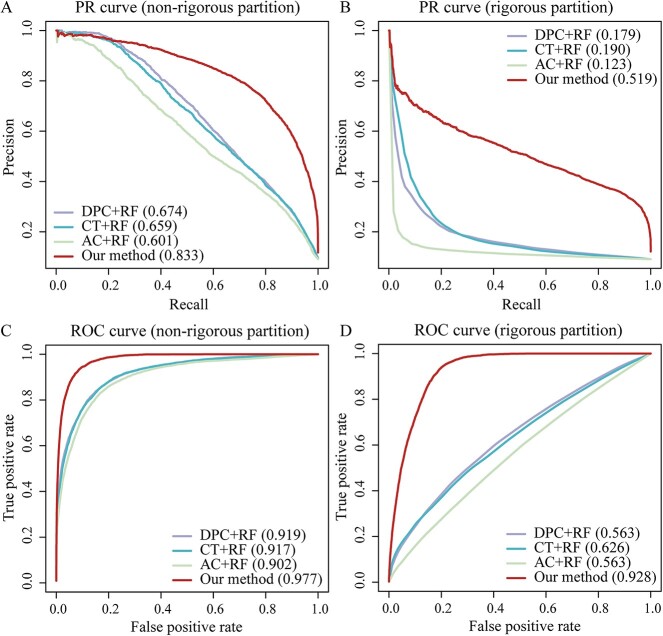
Performance of our multi-modal based models and traditional feature encodings-based RF models in predicting human-herpesvirus PPIs. AUPRC and the AUROC indicated that the multi-modal based LightGBM drastically outperformed DPC + RF, CT + RF and AC + RF. In (**A**), we trained and tested on the non-rigorous dataset while in (**B**) we used the rigorous dataset. (C) ROC curves obtained with the non-rigorous partition dataset and (**D**) the rigorous partition dataset.

We further compared the performance of our proposed method to several traditional feature encodings-based ML approaches, such as RF where we represented the sequences of protein pair samples through DPC, CT and AC feature encodings. Analyses of AUPRCs and AUROCs suggested that our method generally outperformed the routine feature encodings-based RF methods when we considered both the non-rigorous and rigorous partition datasets (Figure 4 and Supplementary Table S1). By introducing more performance measurements in Supplementary Table S1**,** we further quantified the performance of our method in comparison to these traditional feature encodings-based ML models including SVM and MLP, highlighting the substantially better AUROC and AUPRC of our method.

### Performance comparison with existing human–virus PPI prediction methods

To further assess the predictive power of our proposed method, we compared its performance to several existing state-of-the-art human–virus PPI prediction methods based on three different datasets, including a Word2vec encoding-based long-short term memory (LSTM) model [22] and our previous transfer learning method based on CNN [21]. To better present the prediction accuracy and generalization ability of the models, we constructed a novel benchmarking dataset that consisted of 9301 experimentally determined human-herpesvirus PPIs before 2022 (a training set), 138 novel interactions verified in 2022 (an independent test set) and corresponding negative samples (pos-to-neg 1:10). Subsequently, we trained our model on the compiled training set and assessed the corresponding performance using our test set. Moreover, we employed the online webserver of LSTM-PHV (http://kurata35.bio.kyutech.ac.jp/LSTM-PHV) to predict human-herpesvirus interactions in the above independent test set. In particular, we observed that our multi-modal integration method outperformed single-modal based methods and LSTM-PHV utilizing various metrics (Table 2 and Supplementary Table S2), indicating the effectiveness and robustness that the fusion of multi-modal features provides. Furthermore, we compared our multi-modal based LightGBM method to the baseline methods (CT + RF and AC + RF) as well as our previous transfer learning method TransPPI [21] by utilizing the human-herpesvirus PPI dataset of TransPPI. Specifically, the dataset contains 5966 positive human-herpesvirus PPI samples and 59 660 negative samples. Retraining and assessing our multi-modal based LightGBM model through 5-fold cross-validation, we found an obvious improvement of prediction performance with our new method, while our two methods showed balanced precision and recall performance in comparison to the baseline methods (Table 3 and Supplementary Table S3).

**Table 2:** Performance comparison of our multi-modal based LightGBM model with the LSTM-PHV method using compiled human-herpesvirus PPIs as a training set (determined PPIs before 2022) and a test set (determined PPIs in 2022)

Method	AUROC	AUPRC	Precision	Recall	Accuracy	F1-score
Our method^a^	0.919	0.408	0.395	0.688	0.881	0.502
Our method^seq^	0.884	0.298	0.289	0.427	0.858	0.345
Our method^net^	0.820	0.269	0.289	0.427	0.858	0.345
Our method^GO^	0.854	0.348	0.336	0.531	0.867	0.412
LSTM-PHV^b^	0.829	0.387	0.298	0.708	0.829	0.419

^a^Our multi-modal (sequence+network+function) integration method. ^seq^Our sequence-based single-modal method. ^net^Our network-based single-modal method. ^GO^Our function-based single-modal method. ^b^We obtained the prediction results by using the online webserver of LSTM-PHV (http://kurata35.bio.kyutech.ac.jp/LSTM-PHV). In particular, we chose 1097 successfully predicted protein pairs by LSTM-PHV to assess the performance of the LSTM-PHV and our method. We determined our PPIs by utilizing a false positive rate cut-off of 0.1.

**Table 3:** Performance comparison of our multi-modal based LightGBM model with our previous transfer learning method TransPPI on its human-herpesvirus PPI dataset

Method	AUROC	AUPRC	Precision	Recall	Accuracy	F1-score^b^
Our method	0.976	0.859	0.763	0.799	0.959	0.781
CT + RF^a^	0.932	0.737	0.858	0.481	0.946	0.617
AC + RF^a^	0.924	0.699	0.819	0.435	0.940	0.568
TransPPI^a^	0.942	0.768	0.771	0.681	0.953	0.723

^a^Results were retrieved from the original paper of TransPPI [21]. ^b^We used the prediction score threshold 0.5 to determine PPIs.

### Cross-viral subtype prediction test

To further assess and compare the cross-viral subtype prediction ability of our proposed method, we performed a cross-viral taxonomy prediction test based on the human-herpesvirus PPI dataset of DeepViral that was a gene functional and disease phenotype driven CNN method for human–virus PPI prediction [23]. We downloaded the dataset and source codes of DeepViral from the online website (https://github.com/bio-ontology-research-group/DeepViral). In particular, protein interactions that are involved in the HHV-1/HHV-5 subtypes were divided into a validating positive sample set (506 human-HHV-1 PPIs) and a test positive sample set (1241 human-HHV-5 PPIs). Remaining interactions were used as a positive training sample set (3194 PPIs between human and other herpesvirus subtypes excluding HHV-1/HHV-5). As for negative sample selection, we randomly sampled and paired human and herpesviral proteins from positive samples and the human proteome to obtain the negative sample set that was 10 times as large as the positive sample set. Moreover, we also extracted the prediction results of the same dataset (pos-to-neg 1:10) for DeepViral (Supplementary Table S4). We observed that the performance of both our single-modal (sequence, network and function) and multi-modal (sequence+network+function) based methods was obviously better compared to the results we obtained with DeepViral according to various metrics (Table 4), implying that our prediction method had better cross-viral subtype prediction ability. Furthermore, we observed that the prediction results of our sequence-based method and DeepViral showed high sensitivity (recall) and low precision, which may be a consequence of the tendency of the models to predict a large number of interactions. Such an observation also suggested that the multi-modal feature fusion method can improve the sensitivity of the model while keeping accuracy stable.

**Table 4:** Cross-viral subtype prediction performance comparison of our method with DeepViral by using DeepViral’s human-herpesvirus PPI dataset

Method	AUROC	AUPRC	Precision	Recall	Accuracy	F1-score^c^
Our method^a^	0.986	0.896	0.834	0.805	0.968	0.819
Our method^seq^	0.987	0.894	0.720	0.924	0.961	0.809
Our method^net^	0.919	0.554	0.512	0.641	0.912	0.569
Our method^GO^	0.926	0.655	0.765	0.436	0.937	0.555
DeepViral^b^	0.922	0.513	0.292	0.917	0.790	0.443

^a^Our multi-modal (sequence+network+function) integration method. ^seq^Our sequence-based single-modal method. ^net^Our network-based single-modal method. ^GO^Our function-based single-modal method. ^b^We implemented DeepViral using the source codes and human-herpesvirus PPI dataset on Github (https://github.com/bio-ontology-research-group/DeepViral). ^c^We used the prediction score threshold 0.5 to determine the values of precision, recall, accuracy and F1-score.

### Prediction, network and functional analysis of interactions between human and different herpesvirus subtypes

**Figure 5:**
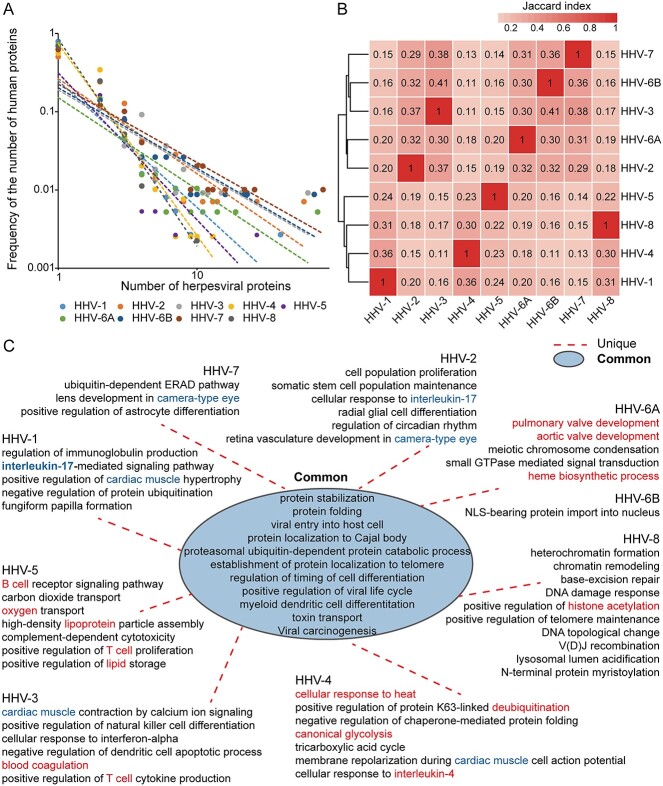
Network topological and functional analysis of predicted viral targets of different herpesvirus subtypes. (**A**) Power laws appeared in the frequency distribution of the number of human proteins that are targeted by a certain number of herpesviral proteins. (**B**) Jaccard indices between targeted proteins of any two herpesvirus subtypes. (**C**) Common and unique enriched functional terms of targeted human proteins of different herpesvirus subtypes (predicted human-herpesvirus PPIs and corresponding confidence scores are available in Table S5).

To predict interactions between human host and nine herpesvirus subtypes (i.e. HHV-1-HHV-5, HHV-6A, HHV-6B, HHV-7 and HHV-8), we trained our three multi-modal based LightGBM models with the non-rigorous datasets. For each herpesvirus subtype, the human-herpesvirus protein pairs among the top 1000 predicted scores of each model were first selected as candidates. Subsequently, we selected the protein pairs with overlapping predictions of any two of the three models as the final high-confidence prediction interactions. Therefore, we predicted 560, 387, 346, 662, 545, 415, 356, 353 and 625 PPIs between human host and proteins of nine herpesvirus subtypes (HHV-1-HHV-5, HHV-6A, HHV-6B, HHV-7 and HHV-8; Supplementary Table S5), respectively, that contain 762 human proteins and 470 herpesvirus proteins in total. By analyzing these targeted human host proteins, we found a power-law distribution of the frequency of the number of human host genes being attacked, suggesting that a majority of human proteins are targeted by one herpesviral protein, while a minority interacts with many herpesviral proteins (Figure 5A). Such an observation is in line with previous findings [51], suggesting the reliability of our model for the identification of novel interactions. To elucidate the similarities and differences in the targeting host patterns of different herpesvirus subtypes, we investigated the Jaccard similarity of targeted human host proteins of different herpesvirus subtypes and performed function and pathway enrichment of our predicted herpesviral targets. Specifically, we found that HHV-1/HHV-4/HHV-5/HHV-8 and HHV-2/HHV-3/HHV-6A/HHV-6B/HHV-7 clustered together, respectively (Figure 5B). In particular, previous studies found that HHV-7 differs from all known human herpes viruses, and although its homology with HHV-6 is small, the two are most closely related to each other [52], which is consistent with our clustering.

Regarding functions/pathways, we observed several commonly enriched functional and pathway terms such as protein stabilization, viral entry into host cell, positive regulation of viral life cycle, toxin transport and viral carcinogenesis, which were shared terms by most of herpesvirus subtypes (Figure 5C and Supplementary Tables S6 and S7). Moreover, we found several related functions and pathways enriched with targeted genes of multiple herpesvirus subtypes such as the interleukin-17 associated biological process that was simultaneously observed in the targets of HHV-1 and HHV-2. Notably, HHV-1 and HHV-2 are both herpes simplex viruses belonging to the *Alphaherpesvirinae* family [7], indirectly confirming the reliability of our predictions. Targets of HHV-2 and HHV-7 were enriched in camera-type eye associated biological processes while HHV-2 was found associated with keratitis and conjunctivitis, suggesting that HHV-7 infection may also be associated with eye disease. Furthermore, cardiac muscle related pathways were observed in target enrichments of HHV-1, HHV-3 and HHV-4. In particular, we also found several unique function and pathway enrichments of different herpesvirus subtypes, such as fungiform papilla formation (HHV-1), radial glial cell differentiation (HHV-2), blood coagulation (HHV-3), cellular response to heat (HHV-4), oxygen transport/high-density lipoprotein particle assembly (HHV-5) and pulmonary valve development/heme biosynthetic process (HHV-6A), which provides clues to the specific infection mechanism of different herpesvirus subtypes for further mechanism analysis.

**Figure 6:**
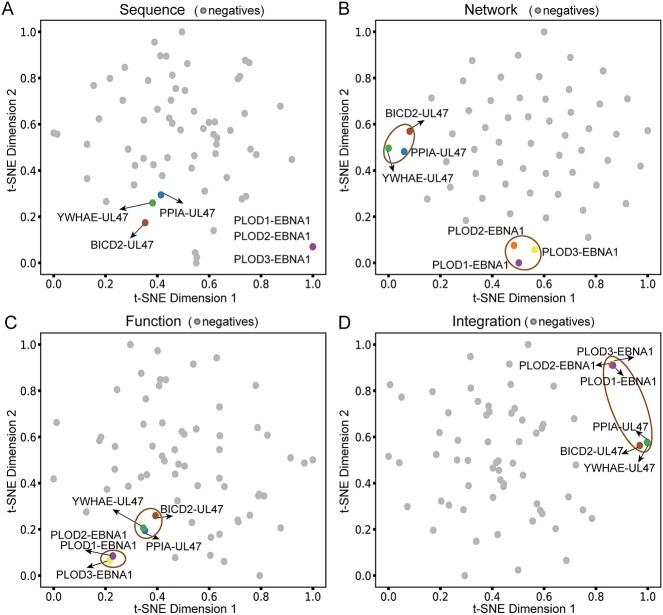
Visualization of single-modal and multi-modal features of six experimentally validated interactions learned from embedding models. (**A**) Sequence features. (**B**) Network features. (**C**) Functional features. (**D**) Multi-modal fusion features.

### Predicted and experimentally confirmed interactions

Conducting a literature search to further assess the reliability of our prediction method, we found experimental corroboration of six predicted human-herpesvirus interactions. Specifically, Dheekollu *et al*. performed FLAG-affinity purification and LC-MS/MS analysis of FLAG-EBNA1 associated proteins, indicating that EBV protein EBNA1 interacts with the PLOD family of proteins (PLOD1, PLOD2 and PLOD3) [53]. Bogdanow *et al*. employed crosslinking mass spectrometry and quantitative proteomics to derive spatially resolved human-CMV and CMV-CMV interactions [54]. Furthermore, we predicted two out of three host–CMV interactions of the CMV protein UL47 (i.e. BICD2-UL47, PPIA-UL47 and YWHAZ-UL47) that were experimentally determined. Interestingly, four PPIs (i.e. PLOD1-EBNA1, PLOD2-EBNA1, PPIA-UL47 and YWHAZ-UL47) were identified by our method under the false positive control rate of 1% (Supplementary Table S8), indicating the reliability of our predictions to some extent.

To further explore the biological significance of our proposed model, we also investigated feature importance and visualized the single-modal and multi-modal features of these six experimentally validated cases (Figure 6). Specifically, we performed t-SNE by feeding our sequence network, functional and merged features of the six experimentally validated interactions and randomly sampled 60 negative samples (not in the training set), respectively. In general, we found relatively large spatial distances between the six interactions and negative samples in the t-SNE plot of ‘Integration’, suggesting that multi-modal fusion features played important roles in distinguishing these interactions from negative samples (Figure 6). Such observations indicated the effectiveness and advantages of our multi-modal model.

## DISCUSSION AND CONCLUSION

Identification of human-herpesvirus PPIs is critical for our understanding of the pathogenic mechanisms of herpes viral infections. While AI/ML-driven prediction of host–virus PPIs has continuously been a hot topic in the field of computational biology, traditional feature encodings-based ML models are susceptible to bias in the training and testing dataset. Specifically, the performance of these models is often overestimated by using data sets that are not rigorously divided (e.g. there are certain shared or similar protein components between the training and test sets). Therefore, the ability of the model to predict new interactions and cross-herpesviral subtypes cannot be fully evaluated using only traditional data sets. In this work, we constructed both datasets of randomly non-rigorous and rigorous samplings, that allowed us to comprehensively assess the host–virus prediction ability of models for known and unknown viral proteins. In particular, the latter rigorous samplings followed the strategy that herpesvirus proteins of both negative and positive test sets were allowed to be sequence-similar to herpesvirus proteins of negative training sets but were obligate to be sequence dissimilar to any herpesvirus proteins in the positive training sets. Such rigorous dataset partition can provide more meaningful results for models to deal with novel herpesvirus proteins and to perform cross-herpesviral subtype predictions.

By using non-rigorous and rigorous benchmarking datasets, we introduced a multi-modal (sequence, network and function) based LightGBM method to predict human-herpesvirus PPIs. With the development of NLP-driven embedding techniques, multi-modal protein features can be effectively obtained, providing more robust information to predict PPIs. We first transformed protein sequences, intra-species PPI network graphs and GO-protein comprehensive network graphs to fixed-dimensional multi-modal feature vectors by utilizing document embedding and graph embedding methods. Subsequently, we trained the single-modal and multi-modal integration models by using a robust ML algorithm, LightGBM. In particular, we employed a novel k-mer extraction method that significantly improved the performance of the sequence-based single-modal model (Table 1 and Figure 2), effectively capturing the semantic features of each amino acid and the whole sequence. Besides, graph embeddings represented network and functional properties of human and herpesviral proteins. In particular, the multi-modal model provided more balanced precision and recall compared to single-modals. In comparison with several traditional feature encodings-based ML methods, our method has exhibited strong robustness and highly balanced precision/recall rates, considering our challenging training and testing datasets. We further compared our method to three existing state-of-the-art human–virus PPI prediction methods and performed a cross-viral prediction test based on their datasets and a newly compiled dataset. The results of our method showed more advantageous and robust performance for both cross-herpesviral subtype prediction and prediction based on the new dataset. Such two datasets were also relatively rigorous with different distribution of training sets and test sets, which provided valuable assessments. Finally, we predicted interactions between human host and different herpesvirus subtypes based on our models. Network and functional analysis of our predicted targets of various herpesvirus subtypes indicated the reliability of our prediction and provided common, related and unique enriched functions/pathways of targets of different herpesvirus subtypes.

Although ML-based human–virus PPI prediction methods have been intensively developed in recent years, they still suffer from several difficulties and limitations. Specifically, the generalization ability of existing methods is still insufficient, which is reflected by sharply dropping accuracy rates when interactions of unseen proteins in the training set were predicted. Furthermore, the selection of negative samples remains a challenging issue, which potentially affects both the prediction accuracy and the generalization ability of the predictive models. In general, new features of proteins can improve the performance of predictive models. For example, the advent of AlphaFold2 [55] allows a reliable prediction of protein structures that can be integrated into models to predict potential interactions between human and viral proteins. Specifically, such protein structures can be easily converted into residue-level structural graph features to be utilized in downstream prediction models. Moreover, large protein language models such as ESM and ProGen have been applied in various protein bioinformatics prediction tasks [56–58]. Such language models are generally generated from very deep neural networks with billions of parameters based on the transformer architectures and trained on millions of protein sequences. These models are powerful in learning protein sequence patterns across evolution, implying that they can be tapped to improve the prediction of human–virus PPIs.

**Key Points**

- A novel k-mer extraction method (i.e. *k* = 1) of the document embedding encoding significantly improves the model performance for human-herpesvirus PPI prediction.
- By introducing multi-modal (i.e. sequence, network and function) embedding feature encodings, we propose a LightGBM model for human-herpesvirus PPI prediction.
- Our method shows superior performance compared to other computational frameworks as well as several existing human–virus PPI prediction methods by utilizing various benchmarking datasets including a cross-viral subtype dataset.

## Supplementary Material

BIB_Supplementary_Tables_bbae005

## Data Availability

The source code is available in GitHub, at https://github.com/XiaodiYangpku/MultimodalPPI/.

## Author Biographies

**Xiaodi Yang** is a research assistant at the Department of Hematology, Peking University First Hospital. Her current research interests include protein bioinformatics, machine learning and NGS analysis.

**Stefan Wuchty** is an associate professor at the Department of Computer Science and Biology, and a member of the Institute of Data Science and Sylvester Comprehensive Cancer Center at the University of Miami. His research interests revolve around systems and network biology.

**Zeyin Liang** is an associate professor at the Department of Hematology, Peking University First Hospital. Her current research interests include immunology and hematology.

**Li Ji** is a technician in charge at the Department of Hematology, Peking University First Hospital. Her current research interests include immunology and hematology.

**Bingjie Wang** is a deputy chief physician at the Department of Hematology, Peking University First Hospital. Her current research interests include immunology and hematology.

**Jialin Zhu** is a physician at the Department of Hematology, Peking University First Hospital. Her current research interests include immunology and hematology.

**Ziding Zhang** is a professor at College of Biological Sciences, China Agricultural University. His research interests are protein bioinformatics and systems biology.

**Yujun Dong** is a chief physician at the Department of Hematology, Peking University First Hospital. His current research interests include immunology and hematology.
